# Impact of the COVID-19 Pandemic on Brazilian Head and Neck Surgery Centers^⋆^

**DOI:** 10.1016/j.bjorl.2023.01.002

**Published:** 2023-01-28

**Authors:** Gustavo Fernandes de Alvarenga, Ana Kober Nogueira Leite, Carlos Neutzling Lehn, Rogério Aparecido Dedivitis, Marianne Yumi Nakai, Beatriz Godoi Cavalheiro, Gilberto Vaz Teixeira, Rafael De Cicco, Luiz Paulo Kowalski, Leandro Luongo de Matos

**Affiliations:** a Universidade de São Paulo, Faculdade de Medicina, Departamento de Cirurgia de Cabeça e Pescoço, São Paulo, SP, Brazil; b Universidade de São Paulo, Faculdade de Medicina, Instituto do Câncer do Estado de São Paulo (ICESP), Departamento de Cirurgia de Cabeça e Pescoço, São Paulo, SP, Brazil; c Hospital do Servidor Público Estadual, Departamento de Cirurgia de Cabeça e Pescoço, São Paulo, SP, Brazil; d Hospital Ana Costa, Departamento de Cirurgia de Cabeça e Pescoço, Santos, SP, Brazil; e Irmandade da Santa Casa de Misericórdia de São Paulo, Departamento de Cirurgia de Cabeça e Pescoço, São Paulo, SP, Brazil; f Instituto Brasileiro de Controle do Câncer, Departamento de Cirurgia de Cabeça e Pescoço, São Paulo, SP, Brazil; g Centro de Pesquisas Oncológicas, Departamento de Cirurgia de Cabeça e Pescoço, Florianópolis, RS, Brazil; h Universidade Federal de Santa Catarina, Faculdade de Medicina, Departamento de Cirurgia, Florianópolis, RS, Brazil; i Instituto do Câncer Doutor Arnaldo Vieira de Carvalho, Departamento de Cirurgia de Cabeça e Pescoço, São Paulo, SP, Brazil; j Faculdade Israelita de Ciências da Saúde Albert Einstein, São Paulo, SP, Brazil

**Keywords:** COVID-19, Pandemics, Cancer treatment, Head and Neck Neoplasm

## Abstract

**Objective:**

The outbreak of the COVID-19 pandemic had a considerable impact on the healthcare access, treatment, and follow-up of oncologic patients. The aim of this study was to evaluate how the COVID-19 pandemic has affected consultation and follow-up demand as well as treatment volume at Brazilian Head and Neck Surgery centers.

**Methods:**

An anonymous online questionnaire was used for collection of data across all Brazilian Head and Neck Surgery Centers across a 3-month period (April‒June 2021). This information included the characteristics of each center, and the perceived self-reported impact of the COVID-19 pandemic on academic activities, residency training, and the diagnosis, treatment, and follow-up of patients with Head and Neck diseases between 2019 and 2020.

**Results:**

The response rate across the 40 registered Brazilian Head and Neck Surgery Centers was 47.5% (*n* = 19). The data showed a significant reduction in the total number of consultations (24.8%) and number of attending patients (20.2%) between 2019 and 2020. The total number of diagnostic exams (31.6%) and surgical procedures (13.0%) conducted over this period also decreased significantly.

**Conclusions:**

The COVID-19 pandemic had a significant national impact on Brazilian Head and Neck Surgery Centers. Future studies should examine the long-term effects of the pandemic on cancer treatment.

**Level of evidence:**

Evidence from a single descriptive study.

## Highlights

• The total number of diagnostic exams and surgical procedures had decreased significantly.
• Surgery for head and neck cancer treatment increased in the total surgical logbook.
• Most residents faced reduction in surgical logbook and deployment to COVID-19 facilities.

## Introduction

The COVID-19 pandemic presents a new challenge to global healthcare, with more than 273 million confirmed cases and 5.3 million deaths worldwide as of December 2021^1^. Brazil was one of the countries most affected by COVID-19, and became an epicenter of the pandemic, with the third greatest number of cases and the second highest mortality rates in the world^2^.

In addition to the treatment and prevention of COVID-19 transmission, healthcare systems are expected to deal with the burden imposed by chronic non-communicable diseases such as cancer, with many patients finding that their needs were left unmet because of the pandemic. The overall impact of the COVID-19 pandemic on oncologic care remains unclear. However, its effects on general healthcare access are expected to be similar for the treatment and follow-up of oncologic patients^3^.

Several adaptations to health service provision have been put in place to reduce the risk of staff and patient infection to redirect resources to the care of patients with COVID-19^4^, ^5^. The adoption of these protective measures most likely limited the capacity for head and neck surgery services to perform diagnostic exams. In addition, the World Health Organization (WHO) has come up with a recommendation to postpone all elective surgeries worldwide, supported by national and international societies, which might have a significant impact on diagnostic procedures, such as biopsies, and on procedures for the treatment of benign head and neck conditions^7^, ^8^. During the COVID-19 pandemic, the safety of the patient and the head and neck surgery team should have been a primordial concern and several guidelines have been published to guide the surgical team^5^, ^6^.

When consider the patient, factors such as social distancing, restriction of non-essential displacement, as well as the postponement of elective consultations and non-emergency procedures all affect the standard of care for oncological patients^8^. Also, the pandemic has imposed an additional burden over cancer treatment related to the inability to receive medical care due to shortage of operating rooms and intensive care unit beds^4^.

Head and Neck Cancer (HNC) is the sixth most common malignancy worldwide, accounting for 6%–8% of the annual cancer incidence, with a worse prognosis in those presenting with advanced illness^5^, ^6^. COVID-19 imposed additional challenges to the diagnosis and treatment of HNC, since healthcare professionals were at a high risk of developing SARS-Cov-2 due to increased contact exposure during the pandemic. Since patients with HNC are often immunocompromised and have poor nutrition, they are also more vulnerable to developing COVID-19^8^. The decision to refer oncological patients for extensive surgery during the pandemic is also complicated, since surgery performed during the SARS-CoV-2 incubation time is associated with increased mortality^9^, ^10^. On the other hand, delaying treatment initiation in patients diagnosed with HNC, a well-stablished time-dependent disease, is associated with increased morbidity and mortality as well as poor functional recovery^11^, ^12^, ^13^.

In summary, the measures put in place because of the COVID-19 pandemic are thought to have contributed to poor healthcare access and difficulties in the diagnosis and treatment of patients with HNC, particularly in resource-limited settings^5^, ^8^. In this context, determining the impact of the COVID-19 pandemic on healthcare systems is needed in order to plan, reorganize, and optimize the management of patients with HNC.

The aim of this study was to evaluate how the COVID-19 pandemic affected Brazilian Head and Neck Surgery centers, with a focus on consultation and follow-up demand as well as surgical treatment burden. In addition, we looked for an effect on the training of Head and Neck Surgery residents since new challenges have emerged to ensure proficiency in advanced surgical training.

## Methods

An anonymous online survey was created using the SurveyMonkey® platform (SVMK Inc., San Mateo, CA, US). The research team undertook pilot testing, and questions were modified to improve comprehension. In the 3-month study period (April 2020–June 2020), the survey was emailed to all Head and Neck Surgery Centers certified as an Educational Surgical Center by the Brazilian Head and Neck Surgery Society.

The questionnaire collected data on the characteristics of each Head and Neck Surgery center (location, public or private setting, surgical capacity, residency training etc.) as well as the impact of the COVID-19 pandemic on the diagnosis, treatment, and follow-up of patients with HNC. The effect on academic activities and residency training was also examined.

Descriptive statistics were performed on the SurveyMonkey® online platform. Absolute and relative frequencies were reported for the qualitative data, and means, medians, standard deviations (SDs), interquartile ranges (percentile 25 [P25%]; percentile 75 [P75%]) or 95% Confidence Intervals (95% CIs) were used for the quantitative data.

## Results

The response rate among the 40 registered Head and Neck Surgery Centers was 47.5% (*n* = 19). The majority were in large urban centers (90%) and in the state of São Paulo (79%). These were mostly public facilities (63%) located in academic hospitals or cancer centers (85%) (Table 1).

**Table 1:** Head and neck surgery centers characteristics.

Variable	*n* (%)
Location	
Capital City	13 (68.42)
Large City	4 (21.05)
Medium City	2 (10.53)
Type of institution	
Academic Hospital	5 (26.32)
Oncological Center	11(57.89)
General Hospital	3 (15.79)
Origin of patients	
Public	12 (63.16)
Private	4 (21.05)
Both	3 (15.79)
Number of surgeons	
Mean/Range	10 (3–20)
Number of residents	
Mean/Range	7 (0–14)

There was a reduction in the total number of consultations (24.8%) as well as the number of new patients (20.2%) in 2020 compared to 2019 (Table 2).

**Table 2:** Impact on head and neck cancer treatment.

Year	First consultation	Follow up consultation	Diagnostic exams	Surgery
2019	1076	5941	1822	819
2020	889	4488	1202	711
Reduction	24.8%	20.2%	31.6%	13%

**Figure 1:**
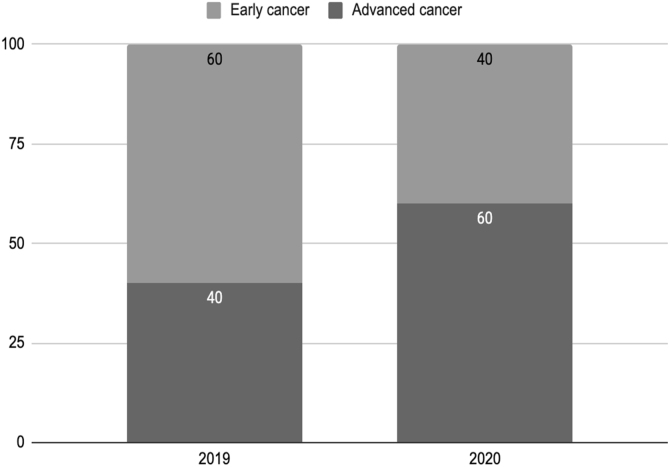
Frequency of patients with early or advanced cancer staging at first consultation by year. In 2019, 60% of patients presented with early cancer (stages 1 and 2) and 40% with advanced cancer (stages 3 and 4) at first consultation. In 2020, 40% of patients presented with early cancer (stages 1 and 2) and 60% with advanced cancer (stages 3 and 4) at first consultation.

The disease stage at first consultation was generally advanced, with an increase of advanced disease at presentation during the pandemic year (Fig. 1). Most centers (60%) adopted telemedicine for consultation during the COVID-19 pandemic, most commonly for follow-up appointments.

**Figure 2:**
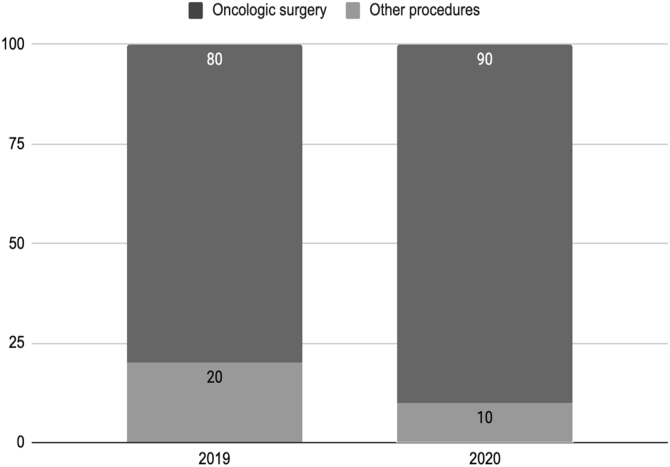
Percentage of cancer treatment surgeries in the total procedures by year. In 2019, surgery for the treatment of cancer represented 80% of the total of surgeries at the respondent head and neck surgery services. In 2020, oncologic surgery represented 90% of the total of head and neck surgeries.

The number of diagnostic exams (31.6%) and the total number of surgical procedures performed (13.0%) also decreased (Table 2), without significance in the waiting time for surgery. The proportion of procedures for cancer treatment in the total surgical logbook between 2019 and 2020 also increased (Fig. 2).

The residency curriculum remained unchanged according to 80% of respondents. The total number of hours dedicated to academic activities also remained the same according to all centers, and virtual meetings or conferences were adopted for clinical discussions in 70% of the institutions.

**Figure 3:**
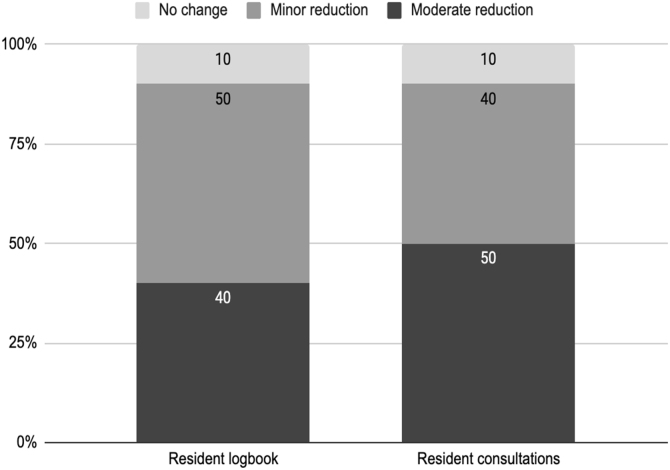
Reported frequency of changes in resident surgical logbook and in resident consultations. Minor changes in the resident surgical logbook were reported by 50% of the head and neck surgery services, while 40% reported moderate changes and 10% no changes. Moderated changes in the resident number of consultations were reported by 50% of them, while 40% reported minor changes and 10% no changes.

In considering the residency workload, 40% of responses indicated no difference between 2019 and 2020. However, when changes were reported, they were equally divided between increasing and reducing (30%). Most respondents (60%) reported that Head and Neck Surgery residents needed to be allocated in exclusive COVID-19 patient facilities (Table 3). Half of Head and Neck Surgery centers experienced a minor reduction (<25%) in the resident surgical logbook, but the same amount of them reported a moderate reduction (<50%) in the number of residents consultations (Fig. 3). Abandonment of residency programs was reported in 30% of respondent centers.

**Table 3:** Impact on head and neck cancer surgery training.

Variable	%
Changes at didactic curriculum	80%
Reduction in academic activities	0%
Changes at workload	
Yes, increasing	30%
Yes, reduction	30%
No	40%
Adoption of video conference	70%
Work at exclusive COVID-19 wards	60%
Centers that had residency abandonment	30%

## Discussion

The first case of SARS-CoV2 in Brazil was identified on 25th February 2020. This completely changed the Brazilian public healthcare system, further compounding the issues of chronic low funding and poor management. The aim of this study was to describe how the COVID-19 pandemic affected the diagnosis, follow-up, surgical waiting times, and oncological education across certified Brazilian Head and Neck Surgery centers between 2019 and 2020^14^, ^15^, ^16^.

Since the public Brazilian Health System provides healthcare to 75% of the population, and cancer treatment is organized towards large specialized public hospitals, the treatment of HNC is performed mostly as a public service at academic hospitals or cancer centers located in large urban areas^18^. These characteristics might have contributed to making Head and Neck Surgery centers highly vulnerable to changes imposed by the COVID-19 pandemic.

A significant reduction in first consultations and total appointments in Head and Neck centers was reported. This might result from the complex interactions between many factors, such as difficulties in reaching the health services due to measures for restriction of populational circulation, patient reluctance, and avoidance of health services because of fear of infection^14^, ^15^. The primary care services were also overwhelmed with COVID-19 cases, imposing a major reduction on elective procedures, and delaying initial diagnosis and referral, thus reducing first consultations at specialty centers. A similar situation was observed for the treatment of benign thyroid conditions, the most common head and neck condition, which might have led to delayed surgical treatment^15^, ^16^.

Responding to the reduction of elective appointments, alternatives to outpatient consultations were adopted, with most of the Brazilian Head and Neck cancer centers using telemedicine for follow-up. However, this practice is relatively new in the country, only being regulated in 2020 due to the COVID-19 pandemic, and its use varies greatly among physicians and services^17^, ^19^. According to this study, the use of telemedicine was restricted to follow-up, most likely reflecting the limitations of the methods used mainly for upper airway examination.

A decrease in the performance of diagnostic upper airway exams was also reported, probably related to the risk of SARS-CoV2 transmission due to aerosol dissemination during upper airway endoscopy^20^, ^21^. Although safety recommendations were put in place to reduce healthcare professional contamination, the adoption of these protective measures most likely limited the capacity for Head and Neck services to perform diagnostic exams^6^, ^15^.

During the period, a nationwide attempt was made to maintain oncological treatment. However, we found a considerable reduction in surgical procedures in almost every respondent center, most likely reflecting diminution of available surgical rooms, intensive care beds, and healthcare professional availability^16^, ^18^, ^23^. Following recommendations of the Brazilian National Sanitary Vigilance Agency and of the National Medical Board, elective procedures were postponed during COVID-19 pandemic, which is demonstrated in our study by an increase in the proportion of procedures for cancer treatment in the total surgical logbook reported by the Head and Neck centers.

In addition, residency programs faced reductions in both clinical activities and surgical cases due to a reduction in elective surgeries, restriction of workforce to essential personnel, and redeployment of residents outside of their specialty to meet COVID-19 related demands for medical care^23^, ^24^. In our study, most respondents reported at least a minor reduction in resident consultations and surgical procedures, reflecting the most common reality nationwide.

Even though academic activities were not suspended or restricted, the COVID-19 pandemic imposed many changes to the didactic curriculum and existing education methods. One of the most evident was the use of technology for remote learning, which was adopted by most of the Brazilian Head and Neck residency programs. The precise impact of the COVID-19 pandemic on education and surgical training is not well-known. However, in certain aspects, especially the use of technology for education purposes, a remarkable new opportunity was presented because of the COVID-19 pandemic^23^, ^24^.

The present study estimated the impact of the COVID-19 pandemic on HNC management and in head and neck surgery training. Unfortunately, the interpretation of our results raised some other questions and lead to some limitations. For example, the main cause of residents training abandon was not accessed. Moreover, the quality of learning by the residents was not accessed, however, the rate of approval by final examination by the Brazilian Head and Neck Society raised in the following years (57.1% in 2019 and, respectively, 75%, 79.6% and 71.9% for 2020, 2021, 2022), accordingly to public results of the Scientific Department. Regarding the HNC treatment, we also do not have data to identify explanations about the reasons to the decrease of the number of treated patients.

## Conclusion

The results of this study should be carefully evaluated. Only half of Brazilian centers responded to the survey. Moreover, our data represents mainly the most affluent and developed part of the country (South and South-East regions) and the impact of the COVID-19 pandemic could be even worse than our findings suggest. Further studies are needed in order to determine the precise effect of the outbreak, especially for the long-term care of patients with cancer.

## Conflicts of interest

The authors declare no conflicts of interest.

## References

[1] Sohrabi C., Alsafi Z., O’Neill N., Khan M., Kerwan A., Al-Jabir A. (2020). World Health Organization declares global emergency: a review of the 2019 novel coronavirus (COVID-19). Int J Surg Lond Engl.

[2] WHO Coronavirus (COVID-19) Dashboard [Accessed 13 January 2022]. https://covid19.who.int.

[3] Jafari A., Rezaei-Tavirani M., Karami S., Yazdani M., Zali H., Jafari Z. (2020). Cancer care management during the COVID-19 pandemic. Risk Manag Healthc Policy.

[4] Wang H., Zhang L. (2020). Risk of COVID-19 for patients with cancer. Lancet Oncol.

[5] Mehanna H., Hardman J.C., Shenson J.A., Abou-Foul A.K., Topf M.C., AlFalasi M. (2020). Recommendations for head and neck surgical oncology practice in a setting of acute severe resource constraint during the COVID-19 pandemic: an international consensus. Lancet Oncol.

[6] Kowalski L.P., Sanabria A., Ridge J.A., Ng W.T., de Bree R., Rinaldo A. (2020). COVID‐19 pandemic: effects and evidence‐based recommendations for otolaryngology and head and neck surgery practice. Head Neck.

[7] Piccirillo J.F. (2020). Otolaryngology Head and Neck Surgery and COVID-19. JAMA.

[8] Brody R.M., Albergotti W.G., Shimunov D., Nicolli E., Patel U.A., Harris B.N. (2020). Changes in head and neck oncologic practice during the COVID-19 pandemic. Head Neck.

[9] Lei S., Jiang F., Su W., Chen C., Chen J., Mei W. (2020). Clinical characteristics, and outcomes of patients undergoing surgeries during the incubation period of COVID-19 infection. EClinicalMedicine.

[10] Lei S., Jiang F., Su W., Chen C., Chen J., Mei W. (2020). Otolaryngology providers must be alert for patients with mild and asymptomatic COVID-19. Otolaryngol Neck Surg.

[11] Graboyes E.M., Kompelli A.R., Neskey D.M., Brennan E., Nguyen S., Sterba K.R. (2019). Association of treatment delays with survival for patients with head and neck cancer. JAMA Otolaryngol Head Neck Surg.

[12] Liao D.Z., Schlecht N.F., Rosenblatt G., Kinkhabwala C.M., Leonard J.A., Ference R.S. (2019). Association of delayed time to treatment initiation with overall survival and recurrence among patients with head and neck squamous cell carcinoma in an underserved urban population. JAMA Otolaryngol Head Neck Surg.

[13] Schutte H.W., Heutink F., Wellenstein D.J., van den Broek G.B., van den Hoogen F.J.A., Marres H.A.M. (2020). Impact of time to diagnosis and treatment in head and neck cancer: a systematic review. Otolaryngol Neck Surg.

[14] Imamura R., Bento R.F., Matos L.L., William W.N., Marta G.N., Chaves A.L.F. (2021). Impact of the COVID-19 pandemic on physicians working in the head and neck field. Int Arch Otorhinolaryngol.

[15] Kowalski L.P., Imamura R., Castro Junior G., Marta G.N., Chaves A.L.F., Matos L.L. (2020). Effect of the COVID-19 pandemic on the activity of physicians working in the areas of head and neck surgery and otorhinolaryngology. Int Arch Otorhinolaryngol.

[16] Melo G.M., Gonçalves A.J., Walder F., Ferraz C., Neves M.C., Abrahão M. (2021 Oct). Analysis of the status of treatment of benign thyroid diseases - a public health problem aggravated in the COVID-19 pandemic era. Braz J Otorhinolaryngol.

[17] Gonçalves S., Kulcsar M.A.V., Matos L.L., Vartanian J.G., Carvalho G.B., Dias F.L. (2020). Overview of care for head and neck cancer cases in Brazilian Cancer Centers during the COVID-19 pandemic. Arch Head Neck Surg.

[18] da Silva M.J.S., O’Dwyer G., Osorio-de-Castro C.G.S. (2019). Cancer care in Brazil: structure and geographical distribution. BMC Cancer.

[19] da Silva R.S., Schmtiz C.A.A., Harzheim E., Molina-Bastos C.G., Oliveira E.B., Roman Rudi (2021). O Papel da Telessaúde na Pandemia COVID-19: Uma Experiência Brasileira. Ciênc Saúde Coletiva.

[20] Workman A.D., Welling D.B., Carter B.S., Curry W.T., Holbrook E.H., Gray S.T. (2020). Endonasal instrumentation and aerosolization risk in the era of COVID-19: simulation, literature review, and proposed mitigation strategies. Int Forum Allergy Rhinol.

[21] Givi B., Schiff B.A., Chinn S.B., Clayburgh D., Iyer N.G., Jalisi S. (2020). Safety recommendations for evaluation and surgery of the head and neck during the COVID-19 pandemic. JAMA Otolaryngol Neck Surg.

[23] Guo T., Kiong K.L., Yao C.M.K.L., Windon M., Zebda D., Jozaghi Y. (2020). Impact of the COVID‐19 pandemic on Otolaryngology trainee education. Head Neck.

[24] Givi B., Moore M.G., Bewley A.F., Coffey C.S., Cohen M.A., Hessel A.C. (2020). Advanced head, and neck surgery training during the COVID-19 pandemic. Head Neck.

