# Supplementary material for: Impact of the COVID-19 Pandemic on Brazilian Head and Neck Surgery Centers
Source: Braz J Otorhinolaryngol. 2023 Jan 28;89(3):456–61. doi: 10.1016/j.bjorl.2023.01.002 (PMC9884113; doi:10.1016/j.bjorl.2023.01.002)
Supplement: Supplementary file 1 [file mmc1.docx]

BJORL-D-22-00220_Supplementary Material

**Supplement** Impacto da COVID-19 nos serviços de cirurgia de cabeça e pescoço.

| Caracterização do serviço de Cirurgia de Cabeça e Pescoço |
| --- |
| 1. Qual o nome do serviço de Cirurgia de Cabeça e Pescoço? |
|  |
| 2. Em qual estado seu serviço está localizado? |
|  |
| 3. Onde está localizado seu serviço? |
| ( ) Capital do estado |
| ( ) Grande centro urbano(>500.000 hab) |
| ( ) Centro urbano (<500.000 hb) |
|  |
| 4. Em qual tipo de instituição o serviço está inserido? |
| ( ) Hospital universitário |
| ( ) Centro de tratamento oncológico |
| ( ) Hospital geral |
|  |
| 5. Qual a principal categoria de pacientes atendidos? |
| ( ) Público (SUS) |
| ( ) Privado (Convênios/Particular) |
| ( ) Ambos |
|  |
| 6. Quantos cirurgiões de cabeça e pescoço atuam no serviço atualmente? |
|  |
| Impacto da COVID-19 no tratamento do câncer de cabeça e pescoço |
|  |
| 7. Qual o número de pacientes atendidos no serviço? |
| a) Ano 2019: |
| b) Ano 2020: |
|  |
| 8. Qual o tempo médio em DIAS de espera entre o diagnóstico e a primeira consulta com cirurgião de cabeça e pescoço? |
| a) Ano 2019: |
| b) Ano 2020: |
|  |
| 9. Na primeira consulta para paciente com diagnóstico de câncer em 2019, quantos pacientes encontravam-se em estágio avançado de doença? |
| ( ) 0‒10% |
| ( ) 11%‒20% |
| ( ) 21%‒30% |
| ( ) 31%‒40% |
| ( ) 41%‒50% |
| ( ) 51%‒60% |
| ( ) 61%‒70% |
| ( ) 71%‒80% |
| ( ) 81%‒90% |
| ( ) 91%‒100% |
|  |
| 10. Na primeira consulta para paciente com diagnóstico de câncer em 2020, quantos pacientes encontravam-se em estágio avançado de doença? |
| ( ) 0‒10% |
| ( ) 11%‒20% |
| ( ) 21%‒30% |
| ( ) 31%‒40% |
| ( ) 41%‒50% |
| ( ) 51%‒60% |
| ( ) 61%‒70% |
| ( ) 71%‒80% |
| ( ) 81%‒90% |
| ( ) 91%‒100% |
| 11. Qual o número de atendimentos ambulatoriais / acompanhamentos realizados? |
| a) Ano 2019: |
| b) Ano 2020: |
|  |
| 12. Houve adoção de telemedicina para atendimento de pacientes durante o período de pandemia por COVID-19? |
| ( ) Sim |
| ( ) Não |
|  |
| 13. Se houve uso da telemedicina, para qual tipo de atendimento foi utilizada? |
| ( ) Primeira consulta |
| ( ) Acompanhamento |
| ( ) Avaliação pré operatória |
|  |
| 14. Qual o número de exames diagnósticos (nasofibroscopia / laringoscopia) realizados? |
| a) Ano 2019: |
| b) Ano 2020: |
|  |
| 15. Qual o número total de cirurgias realizadas? |
| a) Ano 2019: |
| b) Ano 2020: |
|  |
| 16. Qual o tempo médio em DIAS de espera entre a consulta com cirurgião de cabeça e pescoço e o procedimento cirúrgico? |
| a) Ano 2019: |
| b) Ano 2020: |
|  |
| 17. Qual a porcentagem do total de cirurgias realizadas foi para tratamento de câncer em 2019? |
| ( ) 0‒10% |
| ( ) 11%‒20% |
| ( ) 21%‒30% |
| ( ) 31%‒40% |
| ( ) 41%‒50% |
| ( ) 51%‒60% |
| ( ) 61%‒70% |
| ( ) 71%‒80% |
| ( ) 81%‒90% |
| ( ) 91%‒100% |
|  |
| 18. Qual a porcentagem do total de cirurgias realizadas foi para tratamento de câncer em 2020? |
| ( ) 0‒10% |
| ( ) 11%‒20% |
| ( ) 21%‒30% |
| ( ) 31%‒40% |
| ( ) 41%‒50% |
| ( ) 51%‒60% |
| ( ) 61%‒70% |
| ( ) 71%‒80% |
| ( ) 81%‒90% |
| ( ) 91%‒100% |
|  |
| 19. Em seu serviço, houve um protocolo de testagem para COVID-19 de pacientes em pré-operatório? |
| ( ) Sim |
| ( ) Não |
|  |
| 20. Em seu serviço, houve isolamento dos pacientes suspeitos ou confirmados para COVID-19? |
| ( ) Sim |
| ( ) Não |
|  |
| 21. Até o momento, quantos cirurgiões de seu serviço foram infectados por COVID-19? |
|  |
| Impacto da COVID-19 na residência de Cirurgia de Cabeça e Pescoço |
|  |
| 22. Seu serviço conta atualmente com programa de residência médica em Cirurgia de Cabeça e Pescoço? |
| ( ) Sim |
| ( ) Não |
|  |
| 23. Qual o número de residentes atualmente no serviço? |
| ( ) Sim |
| ( ) Não |
|  |
| 24. Houve mudanças na grade curricular da residência? |
| ( ) Sim |
| ( ) Não |
|  |
| 25. Houve adoção de vídeo conferências para atividades acadêmicas? |
| ( ) Sim |
| ( ) Não |
|  |
| 26. Houve redução da carga horária das atividades acadêmicas? |
| ( ) Sim |
| ( ) Não |
|  |
| 27. Houve adoção de videoconferência para atividades de discussão multidisciplinar? |
| ( ) Sim |
| ( ) Não |
|  |
| 28. Houve necessidade de alocação de residentes em áreas não-relacionadas diretamente com a especialidade (ex.: UTI respiratória, enfermaria respiratória)? |
| ( ) Sim |
| ( ) Não |
|  |
| 29. Houve alteração da carga horária dos residentes? |
| ( ) Sim |
| ( ) Não |
|  |
| 30. Qual foi a redução média do volume cirúrgico por residente em relação ao ano anterior? |
| ( ) Não houve mudanças |
| ( ) Diminuição leve (até 25%) |
| ( ) Diminuição moderada (até 50%) |
| ( ) Diminuição importante (até 75%) |
| ( ) Interrupção completa |
|  |
| 31. Qual foi a redução média dos atendimentos ambulatoriais por residente em relação ao ano anterior? |
| ( ) Não houve mudanças |
| ( ) Diminuição leve (até 25%) |
| ( ) Diminuição moderada (até 50%) |
| ( ) Diminuição importante (até 75%) |
| ( ) Interrupção completa |
|  |
| 32. Houve desistências do programa de residência de Cirurgia de Cabeça e Pescoço? |
| ( ) Sim |
| ( ) Não |
|  |
| 33. Até o momento, quantos residentes foram infectados por COVID-19? |
|  |
| 34. Em sua opinião, qual impacto global da pandemia na formação dos residentes? |
| ( ) Positivo |
| ( ) Negativo |
| ( ) Não houve impacto |
